# Evolution of Thiosemicarbazones: From First‐ to Second‐Generation Metal Chelators and Their Reactive Oxygen Species‐Mediated Effects in Melanoma

**DOI:** 10.1002/open.202500333

**Published:** 2026-05-19

**Authors:** Rohina Alim, Scott D. Andrew, Christopher J. Parkinson

**Affiliations:** ^1^ School of Dentistry and Medical Sciences Charles Sturt University Leeds Parade Orange NSW Australia

**Keywords:** melanoma, metal‐chelator, reactive oxygen species, thiosemicarbazide, thiosemicarbazone

## Abstract

Worldwide, melanoma remains a significant public health concern, with an estimated 325,000 new cases and 57,000 deaths recorded in 2020. If current trends persist, these figures are projected to rise to 510,000 new cases and 96,000 deaths annually by 2040, a 50% and 68% increase, respectively. Despite advances in targeted therapies and immunotherapy, there remains an urgent need for novel, selective treatments—particularly potential low cost oral treatments in low and middle income countries. Thiosemicarbazone (TSC) derivatives, particularly those incorporating O—N—S (ONS, first generation) and N—N—S (NNS, second generation) donor motifs, have emerged as promising candidates for melanoma therapy. This review traces the evolution of TSCs from early‐stage compounds to more advanced analogs, emphasizing how structural refinements have enhanced their anticancer potency and selectivity. Classical thiosemicarbazones such as Triapine and Dp44mT display multifaceted mechanisms of action, including potent metal ion chelation, inhibition of ribonucleotide reductase (RR), and the induction of oxidative stress through reactive oxygen species (ROS) generation. The redox activity of their metal complexes facilitates ROS accumulation, which contributes to apoptosis in melanoma cells, that often exhibit elevated basal oxidative stress. Preclinical studies demonstrate strong cytotoxicity and some tumor selectivity for these agents, though clinical translation remains limited. The ongoing development of thiosemicarbazone‐based therapeutics continues to offer promise for more effective and selective melanoma treatment strategies.

## Graphical abstract

Thiosemicarbazones (TSC) have demonstrated promise as new chemotherapeutic agents against melanoma in both in vitro and In vivo studies. TSC are powerful metal coordinating agents, coordinating transition metal ions such as zinc, copper and iron in multiple oxidation states. It is this metal coordination that imparts the anticancer activity. TSC have evolved from a coordination sphere comprising oxygen, nitrogen, and sulfur atoms (eg NT) to nitrogen, nitrogen, and sulfur coordination (eg Triapine, DPC). Several mechanisms of action have been reported, including ribonucleotide reductase (RNR) inhibition, metal ion mobilization and depletion, cyclin‐dependent kinase inhibition and generation of reactive oxygen species.

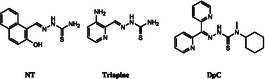

**Abbreviations**
ATP: Adenosine triphosphate
CAT: Catalase
Cu: Copper
Fe: Iron
FlH2: Reduced flavin
FlOx: Oxidized flavin
GPx: Glutathione peroxidase
GSH: Glutathione
GS‐SG: Oxidized glutathione
H_2_0_2_
: Hydrogen peroxide
NADP^+^
: Nicotinamide adenine dinucleotide phosphate
NADPH: Nicotinamide adenine dinucleotide phosphate hydrogen
O_2­_
: Oxygen
O_2­_^.−^
: Superoxide
ROS: Reactive oxygen species
RR: Ribonucleotide reductase
SAR: Structure‐activity relationship
SOD: Superoxide dismutase
TSC: Thiosemicarbazone
UV: Ultraviolet

## 1 Introduction

Melanoma, the deadliest form of skin cancer, exemplifies how the global cancer burden is unevenly distributed, with certain populations facing a dramatically higher risk. Worldwide, melanoma remains a significant public health concern, with an estimated 325,000 new cases and 57,000 deaths recorded in 2020. If current trends persist, these figures are projected to rise to 510,000 new cases and 96,000 deaths annually by 2040, approximately a 50% and 68% increase, respectively [1]. The incidence of melanoma varies markedly by region, reflecting differences in skin type, ultraviolet (UV) exposure, and prevention strategies. For example, incidence rates in Australia and New Zealand are among the highest globally, reaching up to 42 cases per 100,000 people per year, while rates in Asia and Africa are typically below 1 per 100,000 [2].

Australia faces a disproportionate burden of melanoma. In 2023, it was estimated that 18,257 new cases would be diagnosed, making it the third most common cancer in the country. The age‐standardized incidence rate was projected at 69.4 cases per 100,000 persons (85.2 for males and 55.6 for females), a sharp increase from 30 cases per 100,000 in 1982 [3]. The lifetime risk of melanoma in Australia is ≈1 in 17 (7% for males, 4.8% for females) [3]. While advances in early detection and treatment have stabilized mortality rates, melanoma still accounted for an estimated 1,314 deaths in 2023, ranking as the eleventh most common cause of cancer death [3]. The overall burden is intensified by demographic factors such as aging populations and high UV exposure, especially in fair‐skinned populations [4].

A growing body of research has highlighted the critical role of reactive oxygen species (ROS) in melanoma biology. ROS, such as hydrogen peroxide (H_2_O_2_) and superoxide (O_2_^.−^), are naturally produced during cellular metabolism and play a role in cell signaling at normal levels [5]. However, elevated ROS can cause oxidative damage to DNA, proteins, and lipids, contributing to melanoma initiation and progression, particularly with chronic UV exposure [6]. Melanoma cells rely on increased ROS for survival but are also vulnerable to oxidative stress‐induced apoptosis, making ROS a promising therapeutic target [6].

Thiosemicarbazides, specifically thiosemicarbazones (TSC), have emerged as potent anticancer agents. Initially known for inhibiting ribonucleotide reductase (RR), thiosemicarbazones also induce oxidative stress by forming complexes with redox‐active metals like copper (Cu(II)) and iron (Fe(III)). These complexes promote ROS accumulation, which is especially effective against melanoma cells with compromised antioxidant defenses [7].

Structure–activity relationship studies have shown that modifying the thiosemicarbazide core by condensation with aldehydes or ketones to form TSC and further derivatization can significantly improve their selectivity and potency [7]. These derivatives have demonstrated antiproliferative activity both in vitro and in vivo, often with lower toxicity compared to traditional chemotherapeutics. In several models, some metal‐complexed derivatives even outperformed Dinaciclib in efficacy [7]. The versatility of the thiosemicarbazide scaffold and the ability to fine‐tune the redox behavior and selectivity of its derivatives underscore the considerable potential of TSC as next‐generation anticancer agents [8], especially in the context of ROS‐sensitive malignancies like melanoma.

This review examines the pharmacological properties and mechanistic basis of TSC in the context of melanoma therapy, with a particular focus on ROS‐mediated cytotoxicity through metal chelation. It also explores the synthetic versatility of TSC, typically formed via condensation of thiosemicarbazide with aldehydes or ketones, and highlights the importance of continued research and clinical investigation. Given the limited clinical translation to date, there is a clear need for melanoma‐specific studies to fully assess their therapeutic potential.

### 1.1 The Evolution of Thiosemicarbazide and the Discovery of TSC for Targeted Melanoma Therapy

**FIGURE 1:**
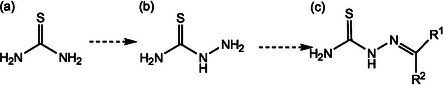
Structural relationships of (a) thiourea, (b) thiosemicarbazide, and (c) thiosemicarbazone (TSC).

Thiosemicarbazide (CH_5_N_3_S) was first recognized for its biological activity in 1944 by Peak and Watson, who published the first study demonstrating the activity of thiosemicarbazide against *Mycobacterium tuberculosis* [9, 10]. This foundational work was later advanced by Domagk in Germany, leading to thiosemicarbazide's introduction as a secondary or adjunct drug for tuberculosis [9, 11]. Structurally, thiosemicarbazide (Figure 1b) features a thiourea core (Figure 1a), with sulfur and nitrogen atoms that confer high nucleophilicity and versatile metal‐binding capabilities [12]. Its N—N—S donor motif is particularly important for forming stable complexes with transition metals, a property that is central to its evolving role in medicinal chemistry [13].

Through condensation with aldehydes or ketones, thiosemicarbazide is transformed into TSCs (Figure 1c), a process that introduces an imine (C=N) bond adjacent to the thiourea moiety while preserving the metal‐chelating N—N—S structure [14]. This transformation greatly enhances the ligand's ability to coordinate metal ions such as Cu(II) and Fe(III), thereby amplifying its biological interactions [14]. TSCs exhibit a wide range of pharmacological activities, including antifungal, antiviral, and anticancer effects, with the latter receiving significant attention due to their ability to interfere with key cancer cell processes [14] and possess ionophoric properties (reversibly bind ions) and are effective in the generation of ROS by improving the redox properties of metal‐ligand complexes [15].

Their anticancer potential arises through multiple mechanisms, including inhibition of RR, disruption of iron and copper homeostasis, and importantly, the induction of oxidative stress via ROS generation [16]. When complexed with redox‐active metals like copper(II) or iron(III), TSCs undergo intracellular redox cycling, producing superoxide anions (O_2_^.−^), hydrogen peroxide (H_2_O_2_), and hydroxyl radicals (·OH). These ROS species can damage DNA, impair mitochondrial function, and trigger apoptosis [16], mechanisms that are especially effective in melanoma cells, which are characterized by inherently high oxidative stress and redox imbalance due to UV exposure and rapid metabolic activity. This redox vulnerability makes ROS‐inducing agents particularly attractive in melanoma therapy [17].

Structure‐activity relationship studies have shown that modifications such as di‐substitution at the terminal N4 position (*N* = *R*_2_ of TSC, Figure 1) and the formation of metal complexes significantly enhance both the potency and selectivity of TSCs against various cancer types, including melanoma [16]. For example, copper(II) and iron(III) complexes of substituted TSC have demonstrated potent antiproliferative activity across various cancer cell lines, including melanoma. In several studies, their efficacy has surpassed that of conventional chemotherapeutic agents, particularly in nonmelanoma cancer models, though promising results have also been observed in melanoma cell lines [18]. Notably, coordination with copper(II) has been shown to enhance the cytotoxic potential of TSCs by several folds compared to their un‐complexed counterparts. Some copper(II) complexes demonstrate IC_50_ values in the low micromolar to sub‐micromolar range in cancer models, highlighting their potency [18]. In a mechanistic investigation, the copper(II) complex VLX60, derived from the TSC VLX50 (3‐(3‐methoxypropyl)‐1‐[[(pyridin‐2‐yl)methylidene]amino]thiourea), exhibited markedly enhanced cytotoxicity relative to the parent ligand. VLX60 showed significant antiproliferative effects across various human tumor cell lines, including melanoma, and was effective in both 2D monolayer and 3D spheroid culture systems [18].

The recognition of TSCs’ efficacy against melanoma is supported by decades of synthetic and biological research, beginning with the seminal work of Brockman and colleagues in 1956, who first reported the antitumor properties of TSC derived from 2‐formylpyridine [19]. Since then, numerous analogs have been synthesized and evaluated on various cancer cell lines, A recent study synthesized and characterized new thiosemicarbazide derivatives, then evaluated their anticancer activity against human melanoma G‐361 cells. The compounds displayed cytotoxicity toward melanoma cells but not normal fibroblasts and induced DNA damage and apoptosis, supporting the ongoing development of thiosemicarbazide‐based agents for melanoma therapy [20] Another investigation synthesized 38 TSC derivatives based on natural product scaffolds (camphene and limonene) and tested them against the SK‐MEL‐37 human melanoma cell line. Several derivatives showed significant antiproliferative activity, and mechanistic analysis confirmed that apoptosis was induced, in part, by ROS generation and caspase activation [21].

These findings confirm that TSCs, though fully synthetic and lacking a singular discoverer, have been developed into targeted anticancer agents through interdisciplinary research focused on their chemical versatility and the central role of ROS generation in exploiting the redox vulnerabilities of melanoma cell.

### 1.2 Emergence and Evolution of TSC in Melanoma Therapy

TSCs have been extensively studied for their anticancer properties, with two distinct generations showing significant activity. The first generation, known as the naphthol (NT) series, features an ONS (oxygen, nitrogen, sulfur) donor set for metal chelation. The second generation, referred to as the pyridyl series, utilizes an NNS (nitrogen, nitrogen, sulfur) chelation motif.

### 1.3 First Generation: O–N–S Donor Motifs and Metal Coordination

**FIGURE 2:**
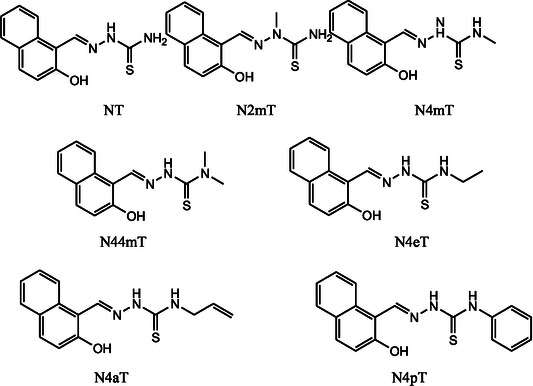
Structures of NT series, sourced from ref. [22].

The ONS chelators (Figure 2), as early generation anticancer TSC, were extensively studied for their efficacy as iron chelators targeting RR. The ligand 2‐hydroxy‐1‐ naphthylaldehyde‐4,4‐dimethyl‐3‐ thiosemicarbazone (N44mT) was the preferred example in the NT series [22].

**FIGURE 3:**
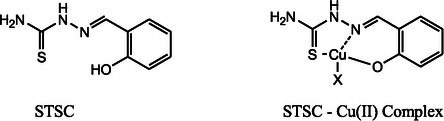
Structures of 2‐hydroxybenzaldehyde (salicylaldehyde) thiosemicarbazone (STSC) and its copper(II) complex. *X* represents a monodentate anion. (from ref. [25, 26]).

N44mT features a structure that combines the 2‐hydroxy‐1‐naphthylaldehyde moiety with a TSC backbone, and its 4,4‐dimethyl substitution enhances both its stability and specificity for iron binding. Acting primarily by chelating intracellular iron (Fe), N44mT deprives RR of the iron necessary for DNA synthesis, resulting in selective cytotoxicity towards cancer cells, which have a heightened demand for iron [22]. While N44mT demonstrated potent RR inhibition and anticancer activity in various cell models, its clinical potential was limited by challenges such as suboptimal bioavailability and systemic stability. These limitations led to the development of more advanced TSCs, such as Dp44mT discussed later, which incorporated additional mechanisms of action to overcome resistance [23, 24]. In the ONS generation, the naphthol (NT, Figure 2) series features a phenolic oxygen donor derived from its naphthol moiety. In contrast, 2‐hydroxybenzaldehyde (salicylaldehyde) TSCs [STSC, Figure 3] also possess a phenolic oxygen, but lack the naphthol structure.

This modification significantly strengthened metal‐binding affinity, including toward copper(II) and iron(III), and improved the capacity of these compounds to generate ROS as part of their anticancer mechanism. For example, studies have shown that TSC exhibit cytotoxicity against various cancer cell lines, attributed to their ability to chelate transition metals and promote ROS‐mediated apoptosis [27]. These findings indicate that the strategic incorporation of a phenolic O—N—S donor set can modulate the redox properties of TSCs and may enhance their tumor‐targeting potential by improving metal coordination and promoting oxidative stress within cancer cells.

### 1.4 Second Generation: N–N–S Donor Motif

The second generation NNS chelators exhibit a superior activity compared to the ONS ligands [28]. The synthesis of compounds featuring the canonical N—N—S donor motif is undertaken through condensation reactions between thiosemicarbazide and various nitrogen‐containing aldehydes or ketones. These reactions produced mono‐substituted TSCs while retaining the thiourea‐based metal‐chelating scaffold, a strategy consistently reported for generating tridentate or bidentate ligands with robust metal‐binding properties [29, 30]. During this period, natural product‐derived frameworks such as camphene and limonene were also often used as molecular backbones, resulting in hybrid molecules that were structurally characterized using standard spectroscopic techniques [21].

**FIGURE 4:**
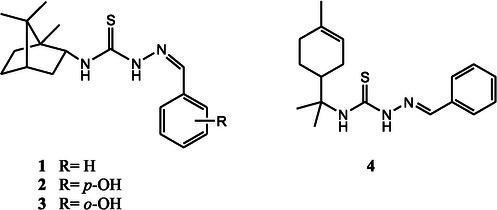
Hhydroxybenzaldehyde–camphor‐based thiosemicarbazones (TSC, structures 1–3), where *R* represents various substituents (e.g., –OH, –H) (b) limonene‐derived TSC (structure 4), which have demonstrated significant activity against the SK‐MEL‐37 melanoma cell line (ref. [21]).

A key objective of this phase was to assess the cytotoxic effects of the synthesized compounds on melanoma cell lines, with a focus on SK‐MEL‐37. Several derivatives demonstrated notable anticancer activity. For instance, compound **3** (Figure 4) (an *o*‐hydroxybenzaldehyde–camphene‐based thiosemicarbazone) emerged as one of the most active, while compound **1** (benzaldehyde–camphene‐based) also showed significant effects. Additional compounds, such as compound **2** (*p*‐hydroxybenzaldehyde–camphene‐based) and various limonene‐derived analogs, including compound **4** (Figure 4), exhibited moderate cytotoxic activity [21]. Mechanistic investigations indicated that these compounds induce apoptosis in melanoma cells, as demonstrated by DNA fragmentation, activation of caspases 6 and 8, and morphological features consistent with programed cell death, including nuclear condensation and cellular shrinkage [18]. Further biochemical analysis implicated ROS as central mediators of this cytotoxic response, with TSC‐metal complexes, particularly those formed with copper and iron, promoting intracellular redox cycling. This process elevates ROS levels, triggering oxidative stress, mitochondrial dysfunction, and apoptotic cell death [21, 29].

This link between TSC activity and ROS is especially relevant to melanoma, which is inherently prone to oxidative stress and metabolic reprograming [31]. TSC exploits this vulnerability by further increasing ROS beyond the cell's threshold, selectively inducing cell death in melanoma cells while sparing normal cells with lower baseline oxidative stress [29, 31].

**FIGURE 5:**
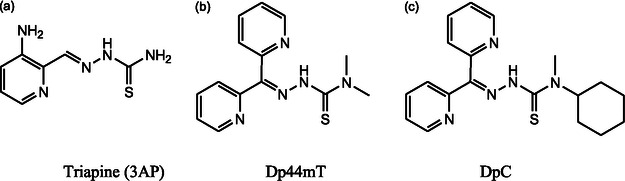
Structures of clinically evaluated TSC (a) Triapine (3AP), (b) Dp44mT, and (c) DpC), sourced from [32].

In parallel with natural product‐based derivatives, pyridyl‐TSC (Figure 5) such as Triapine, Dp44mT and DpC all contain the TSC moiety and have demonstrated strong metal‐binding and ROS‐elevating properties in the context of cancer therapy [23]. Despite their similar core structures, these agents show markedly different efficacy and toxicity profiles, indicating that even subtle structural modifications, such as the cyclohexyl substitution in DpC, can improve therapeutic outcomes or reduce adverse effects. For example, DpC, unlike Dp44mT, does not induce cardiac fibrosis or methemoglobin formation and exhibits improved pharmacokinetics and greater in vivo antitumour activity ‐ even in cancer types that respond poorly to standard treatments [33]. Triapine's TSC moiety enables metal chelation and the formation of redox‐active complexes that can be transported across cell membranes, while Dp44mT, with its *α*‐pyridyl TSC backbone, displays potent RR inhibitory activity and is significantly more potent by ≈50‐ to 100‐fold than Triapine in leukemia cell lines [23]. The primary anticancer mechanisms of these TSCs involve ROS generation via redox cycling of their metal complexes, inhibition of RR, and disruption of metal ion homeostasis, collectively exploiting the redox vulnerabilities of cancer cells [23, 34].

Given that TSCs are designed to chelate metal ions such as iron and copper leading to the generation of ROS via Fenton‐like reactions and ultimately inducing cell death, the role of metal chelation and ROS production is central to their mechanism of action. This is particularly evident in both ONS‐ and NNS‐substituted TSCs, which promote oxidative stress‐induced apoptosis in melanoma cells. Their function in this context is examined in greater detail below.

### 1.5 Thiosemicarbazone Mechanisms of Action Linked to Metal Chelation and Ring Formation

Both ONS (O—N—S donor) and NNS (N—N—S donor) thiosemicarbazones exhibit significant anticancer activity, primarily through their ability to chelate transition metal ions such as iron and copper. This chelation process results in the formation of stable five‐ or six‐membered metallocycles, which are central to therapeutic effects.

In coordination chemistry, five‐membered metallocycles are generally more stable than six‐membered rings due to reduced ring strain and favorable bite angles, resulting in stronger and more persistent metal binding [35]. This increased stability is crucial for medicinal applications, as it enhances the ability of the complexes to interact with biological targets and maintain therapeutic efficacy [35].

**FIGURE 6:**
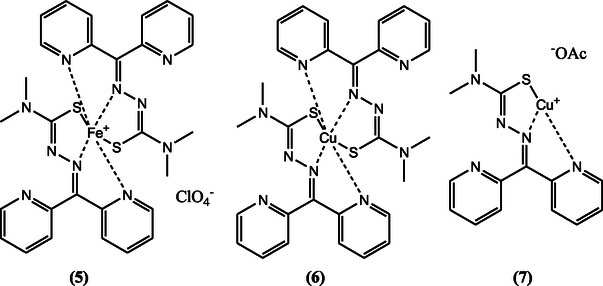
Representative structures of Iron(III) (**5**), Copper(II) 2:1 (**6**) and Copper(II) 1:1 (**&**) complexes of Dp44mT. Redrawn from ref. [36].

For ONS thiosemicarbazones, such as salicylaldehyde thiosemicarbazone (STSC, Figure 6a) the structure includes an oxygen donor, typically from a phenolic group ortho to the imine, which replaces one of the nitrogen donors typically seen in the NNS series. Upon binding metal ions, STSC forms a six‐membered chelate ring involving the oxygen and imine nitrogen and a five‐membered ring involving the imine nitrogen and the sulfur atom of the thiosemicarbazone group. The presence of two distinct metallocycles (5‐ and 6‐membered) contributes to the stability of the metal complex in STSC; however, the 5–5 metallacyclic system is energetically more favorable than that of STSC, as demonstrated by comparative cytotoxic and biochemical studies of thiosemicarbazone ligands and their metal complexes [37].

In contrast, NNS thiosemicarbazones such as Dp44mT (Figure 6b) the 5–5 tridentate coordination mode, binding metal ions via two nitrogen atoms (one from the azomethine group and the other from the hydrazinic nitrogen) and a sulfur atom from the thioamide group. This geometry leads to the formation of two adjacent five‐membered metallocycles. Structural and thermodynamic studies have shown that this dual five‐membered ring system confers even greater stability to the metal complex compared to ONS analogs and enhances both metal sequestration and redox cycling capacity [35]. Dp44mT forms highly stable complexes with copper(II), with a conditional stability constant (log *β*) of 7.08 ± 0.05, which is higher than many ONS‐based chelators tested under similar conditions [36]. For example, studies using spectrophotometric titration and potentiometry have shown that ONS thiosemicarbazones such as 2‐hydroxyacetophenone thiosemicarbazone (an ONS analog) have Cu(II) stability constants (log *β*) in the range of 4.8–5.5 [38, 39]. Additional primary data confirm that salicylaldehyde thiosemicarbazone (STSC), another ONS thiosemicarbazone, forms Cu(II) complexes with log *β* values of 6.26 [40]. These values, while variable, are generally lower or at best comparable to those of Dp44mT, supporting the superior chelation ability of NNS thiosemicarbazones [36].

Therefore, while both ONS and NNS TSC form stable complexes with transition metals, primary data indicate that NNS derivatives such as Dp44mT generally exhibit superior metal chelation strength and complex stability, primarily due to the formation of two adjacent five‐membered metallocycles and their tridentate binding mode [36].

The unique metal‐chelating properties of thiosemicarbazones (TSC), which underpin their ability to modulate redox cycling and ROS production, are discussed in further detail below.

### 1.6 ROS and Their Role in Melanoma

The term ROS refers to chemically reactive molecules containing oxygen produced by an aerobic organism under normal physical conditions [41]. Many of these oxygen species are categorized as radicals because they contain a single unpaired electron (Table 1). For this reason, these oxygen are highly reactive and short lived in biological settings [45].

**TABLE 1:** Major ROS and properties, source, and function. Adapted from refs. [41, 42, 43, 44].

Species	Properties	Source	Function	Antioxidants that reduce them
Superoxide anion (O_2_^−•^)	Good reductant, poor oxidant	Mitochondrial membrane electron transport chain (ETC) and peroxisomes	Promote hydroxyl radical formation	Superoxide dismutase (SOD) Glutathione, Flavonoids, and Vitamin C
Hydroxyl radicals (HO^•^)	Extremely reactive (addition, abstraction, and electron transfer reactions)	Fenton reaction with metal ions (Fe^+2^ or Cu^+^) and peroxisomes	Strongly reacts with DNA, proteins, lipids and carbohydrates and causes severe damage	Vitamin C, Glutathione, Flavonoids and Lipoic acid
Hydrogen peroxide (H_2_O_2_)	Oxidant, but reactions with organic substrates are sluggish. High diffusion capability	Dismutation reaction catalyzed by SOD, peroxisomes and endoplasmic reticulum	At low concentration can cause cellular damage by producing hydroxyl radical. At high concentration can inactivate cellular energy producing enzymes such as glycer‐aldehyde ‐3‐phosphate dehydrogenase	Vitamin C and E, Glutathione peroxidase, Flavonoids, Lipoic acid, Beta carotene and Co Q10
Singlet oxygen (^1^O_2_)	Powerful oxidizing agent with half‐life 10−6 s	Activation of neutrophils and eosinophils as well as reactions catalyzed by lipoxygenases, dioxygenases or lactoperxidase	Can cause DNA and tissue damage	Vitamin A and E
Perhydroxyl radicals (HO_2_^•^/HOO^•^)	Stronger oxidant and more lipid soluble than superoxide. May initiate lipid peroxidation	Protonation of superoxide in the cytosol (at low pH)	Initiate fatty acid peroxidation and promote tumor development	Vitamin E
Peroxyl radical (ROO^•^)	Low oxidizing ability relative to HO•, but great diffusibility	Reaction of molecular oxygen with lipid peroxyl radical (LOO^•^)	DNA and Protein damage	Vitamin C and E
Alkoxy radical (RO^•^)	Intermediate in their reactivity with lipid between ROO• and HO•	Intermediates of any hydrocarbon oxidation	Induces Protein oxidation	RSH antioxidants (*N*‐acetylcysteine, glutathione)

In all aerobic organisms, generation of oxygen species as free radicals is spontaneous because they are the by‐products of aerobic respiration. Some ROS are generated by the mitochondria due to dependence on oxidative processes [44]. The mitochondria are the major producer of endogenous ROS due to its primary role in the production of adenosine triphosphate (ATP) in the ETC where a reduction of molecular oxygen (O_2_) occurs and the superoxide radical (O_2_^.−^) is produced [46]. ROS play a dual role in biological settings. On one hand, when maintained at low to moderate concentrations, they support various biological activities. On the other hand, high levels of ROS within a cell can be detrimental to the cell, resulting in cell death. It is critical, therefore, for cells to maintain ROS levels within a tightly regulated range for normal functioning (homeostasis), because persistent build‐up of ROS can produce oxidative damage [44].

One of their key regulatory roles is involvement in intracellular signaling cascades across multiple cell types, including fibroblasts, endothelial cells, and cardiac myocytes [47]. ROS in low concentrations in the cells can, therefore, act as mitogens to promote cell proliferation and survival. Intermediate concentrations, however, can result in either momentary or permanent cell cycle arrest and also induce cell differentiation [44].

**FIGURE 7:**
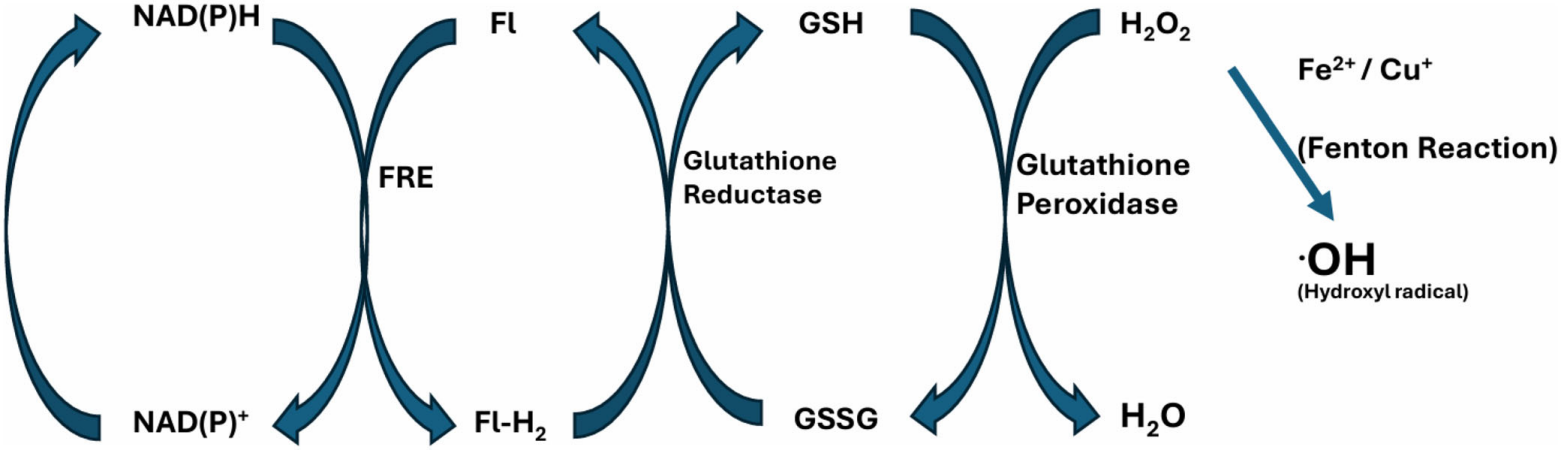
Schematic representationfor the generation of the Hydroxyl radical via the Fenton reaction and the removal of ROS as a result of flavin co‐factor (Fl) dependent glutathione regeneration. (FRE is a flavin reductase enzyme, Fl‐H_2_ is the reduced form of the flavin).

To combat the detrimental effects of ROS, cells utilize a network of antioxidant mechanisms, such as superoxide dismutase (SOD), catalase (CAT) and the glutathione system, to aid in balancing many ROS and to protect their cellular structures [47]. Conversion of ROS into harmless compounds by various antioxidants is a multistep process [48]. Glutathione, as an example, (Figure 7) requires the reduction of nicotinamide adenine dinucleotide (NAD^+^) or the phosphate (NADP^+^) to nicotinamide adenine dinucleotide hydrogen (NADH) and which transfers its hydride via a flavin reductase enzyme to reduce oxidized flavin (FlOx) into FlH_2_. The reduced flavin (FlH_2_) is converted back to oxidized flavin by glutathione reductase enzyme with reduction of oxidized glutathione (GS‐SG) to the antioxidant GSH, which can convert hydrogen peroxide (H_2_O_2_) and other reactive oxygen species into a harmless water molecules [49]. Similarly, GSH can also convert the damaging hydroxyl radical to water.

Like normal cells, ROS in a melanoma cell performs a multiple role in the development of cancer: on one hand, they can promote genetic alterations which are implicated in tumor initiation, growth, and progression. On the other hand, acute elevation of ROS levels within the cancer cell has cytotoxic effects, inducing the stimulation of apoptotic pathways [46]. It is well established that cancerous cells have significantly more oxidative stress compared with noncancerous cells due to their increased metabolic activity, mitochondrial dysfunction, or increased cellular receptor signaling [45]. These cancerous cells adapt to the elevated levels of ROS and up‐regulate antioxidant pathways to balance ROS within their environment. It has been demonstrated that cell death occurs when the normal physiological balance is disturbed and the level of ROS builds up within the cell beyond its capacity, disrupting the normal cellular function, thus triggering cell death [50]. One point of variance between cancerous and noncancerous cells is the amount of further ROS elevation within a cell that can be tolerated, before cell death can occur. Normal cells have lower ROS levels in comparison to cancerous cells and, therefore, a slight increase in ROS levels within a noncancerous cell will be insufficient to cause cell death. The same level of ROS elevation within a cancerous cell will, however, elevate its levels beyond the cell death threshold thus, triggering apoptosis or other forms of cell death [51]. This concept of increasing ROS levels with exogenous agents in cancerous cells beyond their antioxidant protective threshold has shown to be an effective mechanistic approach in the treatment of cancer by TSC through metal‐chelation activity as discussed below.

### 1.7 Role of Transition Metal Ions in ROS Generation

Melanoma cells utilize the abundance of metal ions such as iron or copper already present in their microenvironment to aid with promoting cancer development to further aid prolonging of their survival [52, 53]. Accumulation of metal ions is seen in melanoma cells due to several factors related to their altered metabolism and overexpression of metal transporters and metallothionein proteins that bind and regulate metal ion that are frequently altered resulting in the dysregulation and accumulation for metals [54]. The net effect is that melanoma cells maintain higher intracellular concentrations of metal ions like copper and iron compared to normal cells [55], which not only supports their malignant behavior but also creates vulnerabilities that are exploited by various TSCs targeting metal homeostasis and redox balance.

**FIGURE 8:**
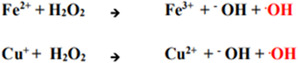
Generation of hydroxyl radical from ferrous and cuprous ions via Fenton reaction.

As illustrated in Figure 7, cells maintain ROS levels with the aid of antioxidants such as glutathione to reduce ROS such as hydrogen peroxide (H_2_O_2_) or the hydroxyl radical into a water (H_2_O) molecule. However, melanoma cells typically exhibit elevated intrinsic ROS levels, as discussed earlier. Coupled with the abundance of redox‐active transition metal ions such as copper and iron in melanoma cells, these metals can interact with endogenous hydrogen peroxide via the Fenton reaction (Figure 8). This reaction produces highly reactive radicals, further increasing intracellular ROS concentrations and contributing to oxidative stress within the cells.

It is crucial to emphasize how exogenous agents such as TSCs exploit the dynamic interplay between transition metal ion concentrations and ROS production in melanoma cells, particularly through modulation of the Fenton reaction. The Fenton reaction (Figure 8) involves the interaction of ferrous (Fe^2+^) or cuprous (Cu^+^) ions with hydrogen peroxide (H_2_O_2_) to generate highly reactive hydroxyl radicals (•OH), which are among the most cytotoxic ROS [48]. These radicals contribute significantly to oxidative stress within cancer cells [48].

TSC are designed to chelate ferric (Fe^3+^) and cupric (Cu^2+^) ion species and these complexes are subject to reduction to produce the ferrous (Fe^2+^) and cuprous (Cu^+^) states, respectively. This redox conversion supports sustained Fenton chemistry by continuously regenerating catalytically active metal ions, thereby enhancing the persistent production of highly reactive hydroxyl radicals [56]. In melanoma cells, where metal ion concentrations are already elevated due to already regulated metal homeostasis, TSC effectively amplify ROS generation to cytotoxic levels. This persistent oxidative stress ultimately overwhelms the antioxidant defense systems, leading to cellular damage and apoptosis [57]. This mechanism of TSC, driven by the disruption of metal homeostasis and sustained oxidative stress, is discussed in further detail below.

### 1.8 The Overall Mechanism of TSCs in Melanoma Cells through Metal Chelation and ROS Generation

**FIGURE 9:**
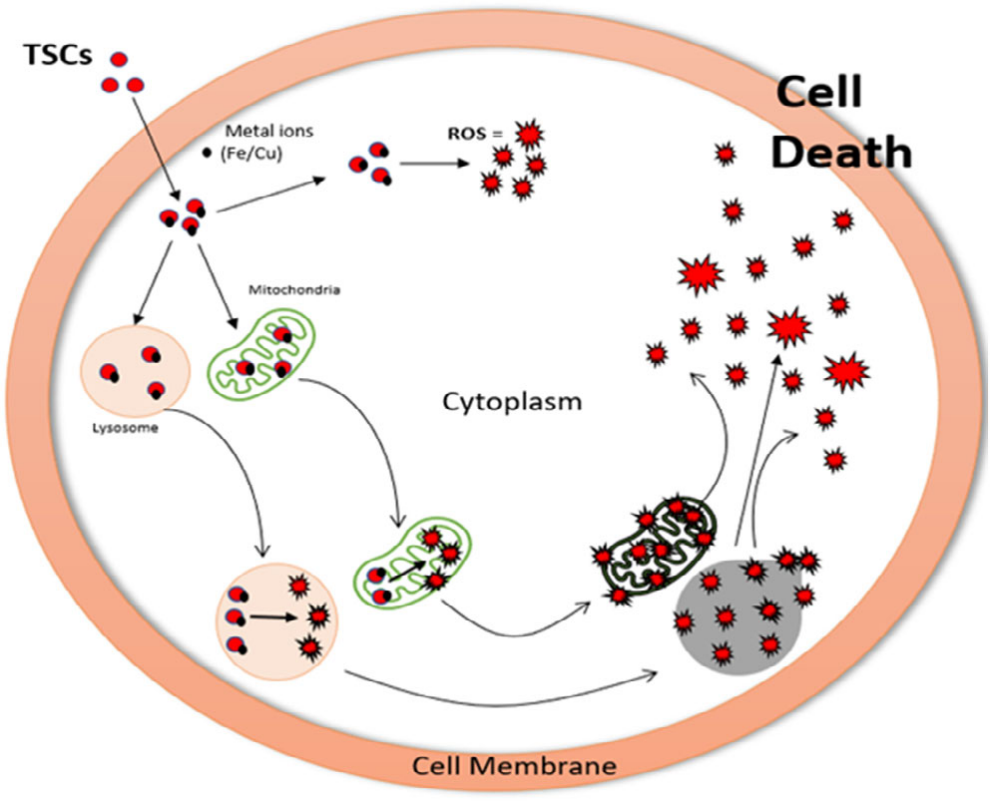
Mechanism of TSC‐induced cancer cell death via metal chelation and ROS generation. TSCs enter cancer cells and chelate intracellular iron and copper ions, particularly in mitochondria and lysosomes. The resulting TSC–metal complexes undergo redox cycling, producing ROS that cause oxidative damage. This leads to mitochondrial dysfunction and lysosomal membrane permeabilization, triggering apoptosis and cell death. Diagram drawn in PowerPoint, information sourced from [15, 58].

TSCs are readily taken up by melanoma cells, where they exert their cytotoxic effects through chelation of redox‐active metal ions like iron and copper. Within the intracellular environment (Figure 9), TSCs may bind these metal ions in the cytosol or localize to organelles with high metal ion concentrations, such as lysosomes and mitochondria. Notably, mitochondria, particularly the ETC and the acidic vesicles like lysosomes are enriched in copper and iron, making them primary targets for metal–TSC complex formation [15, 58, 59].

Once localized, the TSC‐metal complexes undergo redox cycling that catalyzes the generation of ROS, including hydroxyl radicals. The accumulation of ROS rapidly exceeds the cell's antioxidant buffering capacity, resulting in oxidative damage to proteins, lipids, and nucleic acids [15, 58]. This oxidative stress ultimately triggers cell death through pathways such as apoptosis and lysosomal membrane permeabilization [15, 58].

This mechanism is schematically represented in the accompanying Figure 9, which illustrates the cellular uptake of TSCs, their subcellular targeting, and the subsequent ROS‐mediated cytotoxic cascade that culminates in cancer cell death.

### 1.9 Research Gaps and Comparative Efficacy of Thiosemicarbazones in Melanoma Cells

TSCs demonstrate evolving potential in melanoma treatment across successive structural generations, although critical gaps remain in both mechanistic understanding and translational progress.

**FIGURE 10:**
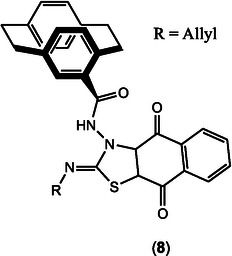
Paracyclophanyl–naphthoquinone hybrid **8**, from ref. [60].

First‐generation ONS‐donor compounds based on the TSC structure, such as the paracyclophanyl–naphthoquinone hybrid **8** (Figure 10), have shown potent in vitro efficacy, achieving complete SK‐MEL‐5 melanoma cell death at 10 μM via CDK1 inhibition (IC_50_ = 54.8 nM) and caspase‐3 activation [60]. Notably, these hybrids outperformed the reference drug Dinaciclib in the same assays [60]. Dinaciclib is a clinically relevant, small‐molecule inhibitor of several cyclin‐dependent kinases (CDKs) notably CDK1, CDK2, CDK5, and CDK9, with low nanomolar IC_50_ values (CDK1: 3–4 nM, CDK2: 1 nM, CDK5: 1 nM, CDK9: 4 nM) [61]. While Dinaciclib is a more potent direct CDK1 inhibitor, the thiosemicarbazone hybrids surpass it in overall cell growth inhibition, likely due to their multimodal mechanisms that include ROS generation and mitochondrial disruption in addition to CDK inhibition [60]. However, these compounds currently lack in vivo efficacy data, and their selectivity toward cancer versus normal cells remains in question.

Second‐generation NNS‐donor pyridyl thiosemicarbazones, such as Dp44mT, expand the mechanistic landscape by disrupting lysosomal membranes and inducing lethal autophagy at 5 μM [24] and have demonstrated significant anticancer activity in vitro and in vivo. Dp44mT exhibits potent antiproliferative effects across a wide range of cancer cell lines, with an average IC_50_ of 0.03 ± 0.01 μM in 28 human tumor cell types, which is markedly lower than that of doxorubicin (average IC_50_: 0.62 ± 0.35 μM) and triapine (average IC_50_: 1.41 ± 0.37 μM). In direct comparison, Dp44mT outperformed doxorubicin in 26 of 28 cell lines tested, including melanoma [62].

In vivo, Dp44mT has shown robust tumor‐suppressive effects in melanoma xenograft models. For example, in SK‐MEL‐28 human melanoma xenografts in nude mice, long‐term treatment with Dp44mT at 0.4 mg/kg/day for 7 weeks reduced the average net tumor size to 49 mm^3^, compared to 612 mm^3^ in control mice, a reduction of over 90% [62]. This dose was well tolerated, with less than 10% body weight loss in mice. At a higher dose (0.75 mg/kg/day), Dp44mT induced even greater tumor regression but was associated with some systemic toxicity (14% body weight loss after 3 weeks). Notably, these antitumor effects were achieved at doses much lower than those required for triapine and without causing systemic iron depletion or overt organ toxicity, distinguishing Dp44mT from other iron chelators [62].

For comparative context, doxorubicin, a standard chemotherapeutic, shows broad cytotoxicity in melanoma cell lines but generally requires higher concentrations to achieve similar levels of cell death [63, 64, 65]. For example, in SK‐MEL‐5 and other melanoma cells, doxorubicin IC_50_ values are typically in the range of 1–5 μM, and the drug is limited by systemic toxicity and the rapid development of resistance in melanoma [63, 64, 65]. In direct comparative studies, thiosemicarbazones (TSCs) such as DpC and Dp44mT have demonstrated either superior activity to doxorubicin (DOX) when tested alone or synergistic effects when used in combination with it. These compounds exhibit lower micromolar IC_50_ values and enhanced selectivity toward melanoma and other tumor cell lines, highlighting their potential as effective anticancer agents [62, 66].

Despite these advances, clinical translation of TSCs in melanoma has stalled. A major reason is the incomplete characterization of their toxicity and therapeutic index; preclinical studies have not yet provided robust data on selectivity for melanoma versus normal cells, nor have they systematically addressed off‐target effects [62, 66]. Additionally, the biological complexity of melanoma, including its adaptive resistance mechanisms and high mutational burden, poses challenges for single‐agent activity [67, 68]. Current clinical trials for melanoma focus on immunotherapies and targeted agents, such as checkpoint inhibitors and adoptive cell therapies, which have demonstrated significant and durable survival benefits in both early‐ and late‐stage disease [69, 70, 71]. In contrast, TSCs have not advanced to clinical trials for melanoma, in part due to the lack of humanized melanoma models, insufficient in vivo efficacy data, and the need for comprehensive toxicity profiling across ONS and NNS chemotypes. For example, recent studies of camphene‐ and limonene‐based thiosemicarbazone derivatives (first generation TSCs) demonstrated promising in vitro antiproliferative activity against melanoma cell lines, but highlighted the necessity for further investigations, including mechanistic studies, and in vivo validation, before advancing toward clinical development [21]. Furthermore, while copper–TSC complexes have shown activity in cisplatin‐resistant melanoma models and complexation with Cu(II) ions can improve antitumor activity against melanoma cells, their broader clinical relevance remains to be established due to limited preclinical testing and the absence of translational studies [72]. Collectively, these gaps highlight the need for further mechanistic studies, improved preclinical models, and rigorous toxicity assessments before TSCs can progress to clinical evaluation in melanoma [21, 72].

In favor of further evaluation, however, is the critical need for cost‐effective new therapies. Recent reports have highlighted the costs of new cancer chemotherapies, the average cost of a agent having escalated beyond the estimated annual cost of $100,000 (USA) in 2014 [73, 74]. Immunotherapies can be around $500,000 *pa*. With the projection that around two‐thirds of new cases will be in low and middle income countries (LMIC) by 2040, it becomes apparent that newer trends in chemotherapeutic development will not be economically feasible in the areas of need [75, 76, 77]. Thiosemicarbazones offer a potential lower cost alternative – the low cost of goods being illustrated by simple preparation and earlier proposals for development in diseases prevalent in LMIC such as malaria (Klaymann, Parkinson) [78, 79]. In LMIC, ease of administration will also have an impact on cost of treatment. Oral bioavailability allows administration outside of a hospital setting. The reported oral bioavailability of several TSC aligns well with this low cost approach [79, 80, 81].

## 2 Conclusion

TSC, particularly those featuring ONS (O—N—S donor motif) and NNS (N—N—S donor motif) frameworks, represent a promising class of anticancer agents with significant potential for melanoma treatment. Their multifaceted mechanisms, including transition metal chelation, CDK inhibition, and the induction of oxidative stress, enable selective targeting of melanoma cells. By binding to redox‐active metals such as iron and copper, TSCs catalyze Fenton‐like reactions, leading to elevated ROS levels. This surge in ROS disrupts redox homeostasis, resulting in DNA damage, mitochondrial dysfunction, and the activation of apoptotic pathways, effects that are particularly pronounced in melanoma cells with already dysregulated oxidative balance.

Recent advancements in TSC chemistry, including the development of water‐soluble analogs and dual‐targeting hybrids, have improved their pharmacokinetic properties, bioavailability, and tumor specificity. These innovations have enhanced the stability of metal complexes and increased the therapeutic potential of TSCs, facilitating better tumor targeting.

Despite these innovations and promising in vitro and in vivo results, such as Dp44mT's submicromolar IC_50_ values and >90% tumor reduction in melanoma xenograft models, significant challenges remain. Comprehensive in vivo efficacy data for ONS‐based agents are still lacking, and direct comparative studies between NNS and ONS chemotypes in melanoma are needed. Moreover, the selectivity of these compounds for cancer cells over normal cells, along with their full toxicity profiles, remains incompletely characterized. The translation of TSCs into clinical trials is further hindered by the absence of humanized melanoma models and the need for more rigorous pharmacokinetic and safety assessments.

Continued preclinical optimization, including systematic structure–activity relationship studies, mechanistic investigations, and the development of advanced animal models, will be essential to address these gaps. Ultimately, extensive clinical trials both in the presence and absence of partnered drugs, supported by multidisciplinary collaboration among chemists, biologists, and clinicians, will be crucial for realizing the full therapeutic potential of TSC‐based therapies in melanoma. With these concerted efforts, TSCs may advance from promising laboratory compounds to effective, targeted treatments for melanoma and, potentially, other malignancies – particularly where the requirement for cost‐effrective treatment is paramount.

## Conflicts of Interest

The authors declare no conflicts of interest.

## Data Availability

Data sharing is not applicable to this article as no new data were created or analyzed in this study.
